# Eu^3+^ Complex-Based Superhydrophobic Fluorescence Sensor for Cr(VI) Detection in Water

**DOI:** 10.3390/nano13182574

**Published:** 2023-09-17

**Authors:** Wei Ding, Sravanthi Vallabhuneni, Jin Liu, Xinzhi Wang, Yue Zhao, Yao Wang, Qinglin Tang, Yanxin Wang, Xiaolin Zhang, Arun Kumar Kota, Jianguo Tang

**Affiliations:** 1 Institute of Hybrid Materials, National Center of International Joint Research for Hybrid Materials Technology, National Base of International Sci. & Tech. Cooperation on Hybrid Materials, College of Materials Science and Engineeeing, Qingdao University, 308 Ningxia Road, Qingdao 266071, China; 2 Department of Mechanical and Aerospace Engineering, North Carolina State University, Raleigh, NC 27695, USA

**Keywords:** electrospinning, fibrous network, hexavalent chromium, inner filter effect, superhydrophobic

## Abstract

Cr(VI) compounds are bioaccumulative and highly toxic pollutants, and there is a need for simple and fast detection methods to monitor their trace levels. In this work, we developed a Eu^3+^ complex-based fluorescence sensor to easily detect Cr(VI) in water droplets. Our sensor consists of a nanofibrous membrane electrospun with a blend of polyvinylidene fluoride (PVDF), silica particles, and Eu^3+^ complex. Upon modifying the membrane surface with fluoroalkyl chemistry, the sensor displayed superhydrophobicity. When a water droplet with Cr(VI) was placed on such a superhydrophobic fluorescence sensor, the overlapping absorption of Cr(VI) and Eu^3+^ complex facilitated the inner filter effect, allowing the selective detection of Cr(VI) down to 0.44 µM (i.e., 45.76 µg L^−1^). We proposed and designed of new inexpensive and fast sensor for the detection of Cr(VI).

## 1. Introduction

Chromium-contaminated liquid waste is a major concern in various industrial sectors like rubber, leather, paper, tanning, sanitary landfills, etc. Chromium compounds, particularly the ones containing hexavalent chromium (Cr(VI)), are bioaccumulative and are highly toxic upon consumption [1,2,3,4,5]. In order to control such adverse effects of chromium on human health, the World Health Organization has set the permissible Cr(VI) concentration level 50 µg L^−1^ in drinking water [6]. Thus, simple, inexpensive, and efficient chromium sensing technologies that can monitor Cr(VI) levels in drinking water have gained significant attention [7,8,9]. While fluorescence sensors based on quantum dots and organic dyes have been explored for Cr(VI) detection, they suffer from limitations such as low sensitivity, short fluorescence lifetimes, broad emission bands, photobleaching, etc. [1,10,11,12,13,14,15,16]. Fluorescence sensors based on the inner filter effect (IFE) using lanthanide complexes overcame these limitations, but they are vulnerable to water [17,18,19,20,21,22,23,24,25,26,27,28,29]. For example, Tan et al. developed Pickering emulsion and quantum dot (QD) doping technology to fabricate Janus silica nanoflake-based fluorescence sensor arrays for the pattern recognition of multiple heavy metal ions [30]. Amin et al. synthesized a novel hydrazone functionality-based spectrophotometric probe for selective and sensitive estimation of toxic heavy metal ions [31]. Melnikov et al. proposed a new fluorescent method to selectively recognize heavy metals in an aqueous solution via employing an array of several fluorescent probes: acridine yellow, eosin, and methylene blue [32]. Currently, the detection methods mainly depend on complicated apparatus, such as liquid chromatography–tandem mass spectrometry [33], surface-enhanced Raman spectroscopy [34], high-performance liquid chromatography coupled with UV-vis determination [35], etc. However, these methodologies are usually limited owing to their drawbacks, including high cost, complicated operation, and time consumed. In contrast to the conventional instrumental methods, luminescent sensing has been proven to be a promising analysis technique owing to its high sensibility, short response time, easy manipulation and low cost. Furthermore, non-contact fluorescence sensing based on IFE has never been investigated with lanthanide complexes for Cr(VI) detection.

In this work, we developed the first ever lanthanide-complex-based non-contact fluorescence sensing approach coupled with IFE to enable Cr(VI) detection in aqueous liquids with high sensitivity and high selectivity. Our Cr(VI) sensor consists of a nanofibrous membrane electrospun with a blend of polyvinylidene fluoride (PVDF), silica particles and Eu(TTA)_3_Phen complex (TTA:2-thenoyltrifluoroacetone, Phen:1,10-Phenanthroline). We chose this Eu^3+^ complex among lanthanide complexes because of its spectral overlap with Cr(VI) to leverage the IFE. We used silica particles to impart appropriate texture and fluoroalkyl chemistry (after surface modification), which together result in superhydrophobicity (i.e., extreme repellency water) [36,37]. Due to the superhydrophobicity, water beads up on our sensor, allowing Cr(VI) detection using droplets with low liquid volumes (3 µL), unlike the traditional Cr(VI) sensors. Our sensor demonstrated Cr(VI) detection at concentrations as low as 0.44 µM (i.e., 45.76 µg L^−1^), with high selectivity against metal cations and anions in water. We anticipate that our results will open prospects for the design of novel inexpensive and reusable Cr(VI) sensors.

## 2. Experimental Section

### 2.1. Synthesis of Eu^3+^ Complex

Eu^3+^ complex was obtained by complexation of EuCl_3_·6H_2_O, TTA, and Phen according to the literature with some modifications [38]. First, 0.05 M solutions of EuCl_3_·6H_2_O, TTA, and Phen in DMF were prepared separately at room temperature under magnetic stirring. EuCl_3_·6H_2_O solution and TTA solution were added with the 1:3 ratio and stirred for 30 min at room temperature. Subsequently, one part of Phen solution was added to the EuCl_3_·6H_2_O and TTA solution and stirred for 2 h at room temperature. Post stirring, the solution appears colorless and contains Eu^3+^ complex dissolved in DMF.

### 2.2. Synthesis of Silica Particles

In this step, 400 nm silica particles (Figure S1) were synthesized via the classic Stöber method with 1 mL TEOS and 50 mL ethanol using 1.17 M ammonia solution [39]. A dispersion of the synthesized silica particles (1.25 g) in 5 mL DMF was prepared using an ultrasonic cell pulverizer (BILON96, Shanghai Bilon Co., Ltd., Shanghai, China) for electrospinning.

### 2.3. Electrospinning Nanofibrous Membranes

**Figure 1:**
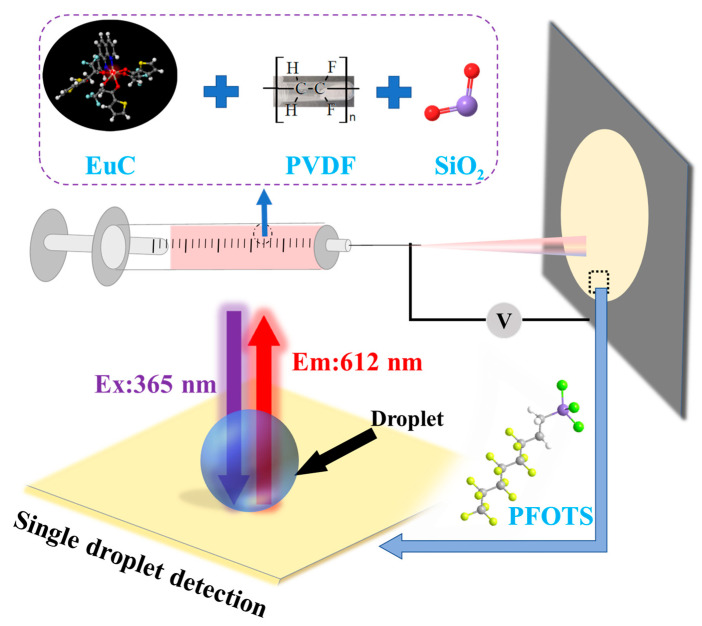
Schematic illustrating the fabrication of our Cr(VI) sensor.

Five nanofibrous membranes (i.e., FM 1, FM 2, FM 3, FM 4 and FM 5) were fabricated via electrospinning using different solutions/dispersions. FM 1 was fabricated by electrospinning Eu^3+^ complex (55.25 mg) only. FM 2 was fabricated by electrospinning PVDF (1.25 g) only. FM 3 was fabricated by electrospinning PVDF (1.25 g) and Eu^3+^ complex (55.25 mg). FM 4 was fabricated by electrospinning PVDF (1.25 g), Eu^3+^ complex (55.25 mg), and silica particles (0.18 g). FM 5 was fabricated by treating FM 4 (Figure 1) with PFOTS at 100 °C for 90 min in an enclosed chamber. A mixture of 10 mL DMF and 3 mL acetone was used as the solvent for all solutions/dispersions. The solutions/dispersions were prepared by stirring the respective constituents at 500 rpm and 40 °C for 6 h. For electrospinning, the solutions/dispersions were loaded into a 5 mL syringe (Chongqing Co., Shanghai, China) with a blunt stainless-steel needle. The solution was fed at a flow rate of 0.3 mL min^−1^ using a syringe pump and was collected on a grounded aluminum foil. A positive DC voltage of 16 kV was applied at the stainless-steel tip and the distance between the stainless-steel tip and collector was set at 16 cm.

## 3. Characterizations

The surface morphology of the nanofibrous membranes was characterized using a scanning electron microscope (SEM; TESCAN, VEGA3, Brno, Czech Republic). The surface chemistry of nanofibrous membranes was characterized using an X-ray photoelectron spectrometer (XPS; VG Scientific, VG ESCALAB 220iXL, Waltham, MA, USA). The water repellency of nanofibrous membranes was characterized by measuring contact angles of water droplets using a contact angle meter (JC2000D). Contact angle measurements were repeated at least four times on each nanofibrous membrane. The photophysical properties of our nanofibrous membranes were characterized using UV-vis spectra and fluorescence spectra. UV-vis spectra of nanofibrous membranes were obtained using a UV755B (Youke, Beijing, China) spectrophotometer at ambient temperature. Fluorescence spectra were obtained using Cary Eclipse Fluorescence spectrophotometer (Varian, Palo Alto, CA, USA) equipped with a 75 kW Xenon lamp (Varian, Palo Alto, CA, USA) as the excitation source. The quantum yield (*Φ_tot_*) and fluorescence lifetime (τ_obs_) measurements were carried out using FLs 980 instrument (Edinburgh Instruments Ltd., Edinburgh, UK). For each nanofibrous membrane, the measurements were repeated at least three times.

### Cr(VI) Sensing with Aqueous Droplets

Cr(VI) sensing with aqueous droplets was conducted on FM 5 (i.e., Cr(VI) sensor) using fluorescence spectra obtained from micro-spectroscopy (CRAIC, San Dimas, CA, USA; Figure S2). Multiple stock solutions were prepared for a wide range of Cr(VI) concentrations by diluting 0.1 M Cr_2_O_7_^2−^ (using K_2_Cr_2_O_7_) solution. A 3 μL droplet from the stock solution was placed over the Cr(VI) sensor, excited using a 365 nm Xenon light source (Varian, Palo Alto, CA, USA) and the fluorescence response was collected. For each droplet concentration, the test was repeated at least two times with identical parameters at room temperature. To evaluate the selectivity of Cr(VI) sensing, different 0.1 M stock solutions with a series of metal cations (Ba^2+^, Ca^2+^, K^+^, Mg^2+^, Mn^2+^, Na^+^) and anions (F^−^, Br^−^, Cl^−^, I^−^, HSO_4_^−^, CH_3_COO^−^) were prepared in distilled water and diluted to the desired concentration, and the fluorescence responses from the droplets were analyzed.

## 4. Results and Discussion

### 4.1. Surface Morphology, Surface Chemistry, and Water Repellency of Cr(VI) Sensor

**Figure 2:**
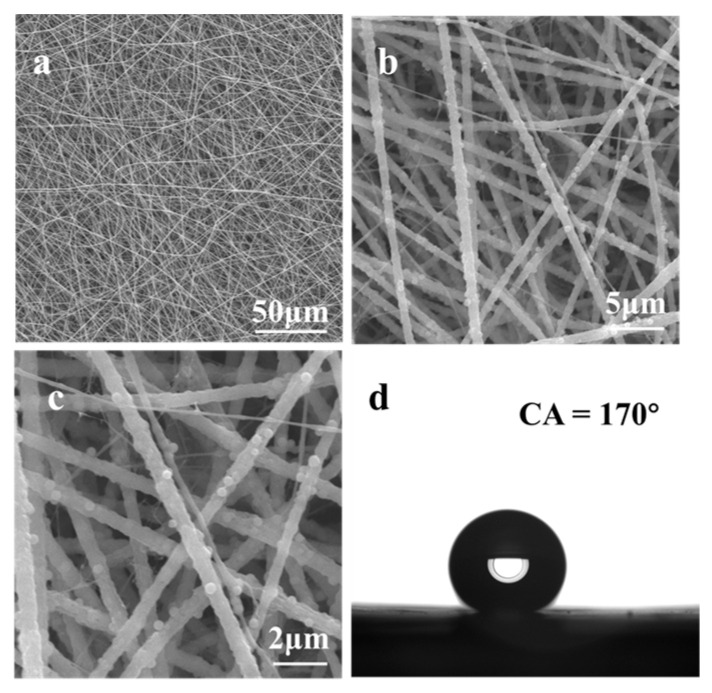
(**a**–**c**) SEM images of our Cr(VI) sensor at different magnifications. (**d**) A 3 μL water droplet beading up on our Cr(VI) sensor.

**Figure 3:**
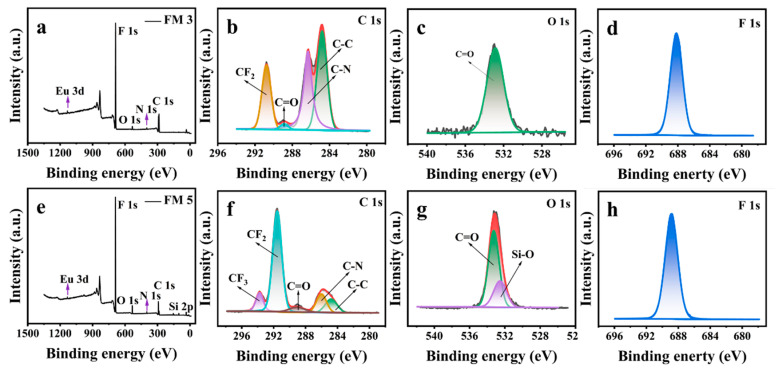
(**a**–**d**) Full survey XPS spectrum, high-resolution C 1s spectrum, high-resolution O 1s spectrum, and high-resolution F 1s spectrum of FM 3, respectively. (**e**–**h**) Full survey XPS spectrum, high-resolution C 1s spectrum, high-resolution O 1s spectrum, and high-resolution F 1s spectrum of FM 5 (i.e., our Cr(VI) sensor), respectively.

Our Cr(VI) sensor consists of a nanofibrous membrane electrospun with a blend of polyvinylidene fluoride (PVDF), silica particles, and Eu(TTA)_3_Phen complex (Figure 1). In order to evaluate the influence of Eu^3+^ complex, silica particles, and fluoroalkyl chemistry on the photophysical properties and water repellency of our Cr(VI) sensor, we fabricated five nanofibrous membranes (see Section 2) by electrospinning: Eu^3+^ complex only (FM 1), PVDF only (FM 2), PVDF + Eu^3+^ complex (FM 3), PVDF + Eu^3+^ complex + silica particles (FM 4), and modifying FM 4 with 1H,1H,2H,2H-Perfluorooctyltrichlorosilane (PFOTS) to impart fluoroalkyl chemistry (FM 5; our Cr(VI) sensor). We characterized the surface morphology of our nanofibrous membranes using scanning electron microscopy (SEM). The SEM images of FM 2 (Figure S3a–c), FM 3 (Figure S3e–g), FM 4 (Figure S3i–k), and FM 5 (Figure 2a–c) indicate a nanofibrous morphology. It is evident that silica particles in FM 4 and FM 5 provide additional texture. After surface modification with PFOTS, FM 5 (i.e., Cr(VI) sensor) displayed superhydrophobicity due to a combination of the texture imparted by silica particles and the low solid surface energy imparted by PFOTS. Figure 2d shows that the sensor displays a water static contact angle of 170° and Movie S1 shows water droplets easily rolling off the sensor at a 5° tilt angle, demonstrating the sensor’s superhydrophobicity. Further, the sensor was also extremely repellent to aqueous droplets containing different concentrations of Cr(VI), and a series of metal cations and anions (Figures S4–S6). We characterized the surface chemistry of our nanofibrous membranes using X-ray photoelectron spectroscopy (XPS). Figure 3 shows a comparison of the XPS spectra of FM 3 (PVDF + Eu^3+^ complex) and FM 5 (PVDF + Eu^3+^ complex + silica particles, and subsequent surface modification; our Cr(VI) sensor). Comparison of the survey spectra (Figure 3a,e) indicates the presence of Eu 3d, F 1s, O 1s, N 1s, and C 1s peaks in both FM 3 and FM 5, and the presence of a Si 2p peak only in FM 5. This implies that PVDF and Eu^3+^ complexes are present in both FM 3 and FM 5, and silica particles are present only in FM 5. Comparison of the high-resolution C 1s spectra (Figure 3b,f) indicates the presence of -CF_2_, C=O, C-N, and C-C peaks in both FM 3 and FM 5, and the presence of a -CF_3_ peak only in FM 5. This indicates the fluorination on FM 5 due to surface modification with PFOTS. Comparison of high-resolution O 1s spectra (Figure 3c,g) indicates the presence of a C=O peak in both FM 3 and FM 5, and the presence of Si-O peak only in FM 5. This implies the presence of silica particles only in FM 5. Comparison of high-resolution F 1s spectra (Figure 3d,h) indicates the presence of a F 1s peak in both FM 3 and FM 5, as expected.

We also characterized the surface composition of FM 5 (i.e., our Cr(VI) sensor) through elemental mapping using energy dispersive X-ray spectroscopy (EDS; Figure S7). EDS elemental mappings indicate the presence of F, Si, Eu, O, N, S, and C in FM 5. This implies the presence of PVDF, silica particles, and Eu^3+^ complex in FM 5. We characterized the composition of nanofibrous membranes using Fourier-transform infrared spectroscopy (FTIR; see Supplementary Materials, Figure S8). The FTIR spectra indicate the presence of a Si-O group (peak at 1100 cm^−1^) only in FM 4 and FM 5. The presence of -CF (peak at 1158 cm^−1^), -CF_2_ (peak at 1450 cm^−1^), and -CF_3_ (peak at 1240 cm^−1^) groups only on the FTIR spectrum of FM 5 imply surface fluorination due to PFOTS. We characterized the melt temperatures of nanofibrous membranes using differential scanning calorimetry (DSC). The DSC curves (Figure S9) indicate that there is no change in the phase and melt temperatures due to incorporation of silica particles and Eu^3+^ complex in nanofibrous membranes. We also determined the phases of PVDF and crystal planes of silica particles using X-ray diffraction (Supplementary Materials, Figure S10).

### 4.2. Photophysical Properties of Cr(VI) Sensor

**Figure 4:**
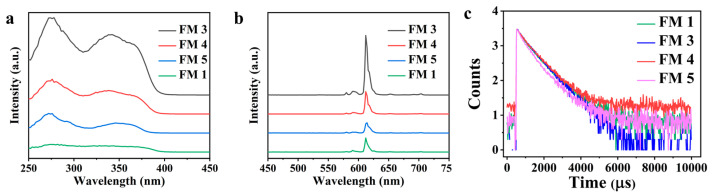
(**a**) The emission spectra of different samples. (**b**) The excitation spectra of different samples. (**c**) Fluorescence lifetime of the samples.

We characterized the photophysical properties of our Cr(VI) sensor (i.e., FM 5) using fluorescence spectra. The fluorescence excitation and emission spectra on all samples containing an Eu^3+^ complex (i.e., FM 1, FM 3, FM 4, FM 5) displayed an excitation peak between 300 nm and 400 nm, which is primarily attributed to the excitation of the Eu^3+^ complex, and a sharp emission peak at 612 nm (Figure 4a), which corresponds to the Eu^3+^ energy level transitions, ^5^D_0_ → ^7^F_J_ (J = 0 at 579 nm, 1 at 590 nm, 2 at 612 nm, 3 at 651 nm, and 4 at 702 nm). All the samples show only one line transition for a non-degenerate electric dipole mechanism. To understand the energy transfer mechanism during fluorescence, we determined the lowest singlet and triplet excitation states of Eu^3+^, TTA, and Phen. The peaks at 23,999 cm^−1^ (417 nm) and 26,616 cm^−1^ (376 nm) correspond to the triplet state (^3^ππ^*^) of TTA and Phen, respectively. The peaks at 27,195 cm^−1^ (368 nm) and 31,080 cm^−1^ (322 nm) correspond to the excited state (S_1_) of TTA and Phen, respectively. The emission energy of ^5^D_0_ for Eu^3+^ lies at 16,352 cm^−1^. The energy difference between ^5^D_2_ of Eu^3+^ (456 nm) and triplet state of the TTA is 4669 cm^−1^, indicating a triplet (T_1_) excitation of TTA to ^5^D_2_ of Eu^3+^ excited triplet (T_1_) by intersystem crossing. The fluorescence intensity depends on the energy transfer resulting from the excitation in Eu^3+^ complex and is a critical parameter that determines the sensitivity. It must be noted that the fluorescence intensity of our Cr(VI) sensor (i.e., FM 5) is lower compared to the other samples (i.e., FM 1, FM 3, FM 4). This is due to the reduced bond coordination between Eu^3+^ ions in Eu^3+^ complex and fluorine atoms of PVDF, interference due to silica particles [40,41], and surface fluorination due to surface modification with PFOTS.

We further investigated the fluorescence properties by evaluating the fluorescence decay and fluorescence quantum yield (*Φ_tot_*) of all samples (i.e., FM 1, FM 3, FM 4, FM 5). The measured fluorescence lifetimes (*τ_obs_*) of FM 1, FM 3, FM 4, and FM 5 were 720, 700, 726, and 626 μs, respectively (Figure 4b and Table 1). Fluorescence quantum yield (*Φ_tot_*) was determined using Equations 1–3 as [13,42]:

(1) $$\phi_{tot}=\phi_{sen}\phi_{Eu}\;$$

(2) $$\phi_{Eu}=\frac{A_{RAD}}{A_{RAD}+A_{NR}}=\frac{\tau_{obs}}{\tau_{RAD}}$$

(3) $$\tau_{RAD}=\frac{1}{{\;A}_{RAD}}=\frac{1}{A_{MD,0}\times n^{3}\times \left(\frac{I_{tot}}{I_{MD}}\right)}\;$$

Here, *Φ_sen_* is the energy transfer sensitization, *Φ_Eu_* is the intrinsic quantum yield, *A_RAD_* is the radiative decay rate, *A_NR_* is the non-reactive decay rate, *τ_RAD_* is the radiative lifetime, A_MD,0_ is the spontaneous emission probability of magnetic dipole (MD) ^5^D_0_ → ^7^F_1_ transition (14.65 s^−1^), and n is the refractive index of the medium (1.42 for PVDF).

Table 1 indicates a lower fluorescence lifetime (*τ_obs_* = 626.30 μs) and a lower quantum yield (*Φ_tot_* = 27.87%) on the Cr(VI) sensor (i.e., FM 5) compared to FM 1 (*Φ_tot_* = 66.02%, *τ_obs_* = 726.32 μs), FM 3 (*Φ_tot_* = 45.61%, *τ_obs_* = 700.84 μs), and FM 4 (*Φ_tot_* = 33.74%, *τ_obs_* = 720.04 μs). This is due to the silica particles and surface fluorination of the Cr(VI) sensor.

**Figure 5:**
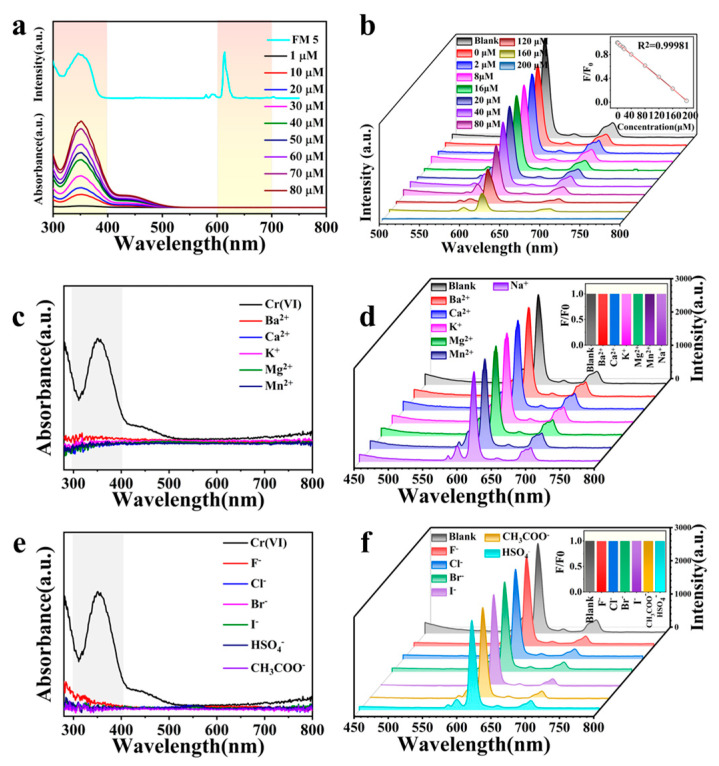
(**a**) Spectral overlap between the excitation of our Cr(VI) sensor (i.e., FM 5) and absorption of aqueous droplets with Cr(VI) at different concentrations. (**b**) Fluorescence quenching on our Cr(VI) sensor with aqueous droplets with different concentrations of Cr(Ⅵ). Inset shows Stern–Volmer plot of the sensor quenched by Cr(VI) aqueous droplets. (**c**,**d**) The UV-Vis absorption spectra and fluorescence response, respectively, from aqueous droplets with different metal cations at 0.1 M concentration. (**e**,**f**) The UV-Vis absorption spectra and fluorescence response, respectively, from aqueous droplets with different anions at 0.1 M concentration. Insets in (**d**,**f**) show F_0_/F = 1, indicating no discernible effect in fluorescence quenching.

We investigated the sensitivity of our Cr(VI) sensor by detecting Cr(VI) in aqueous droplets for different Cr(VI) concentrations (i.e., 1 μM to 80 μM). Figure 5a shows an overlap between the excitation spectrum of Cr(VI) sensor (i.e., FM 5) and absorption spectrum of Cr(VI) at different concentrations. It also illustrates that the increasing concentration of Cr(VI) in aqueous droplets results in increasing absorption intensities. Consequently, the emission intensities of our Cr(VI) sensor are suppressed with increasing Cr(VI) concentrations in aqueous droplets. This is referred to as fluorescence quenching [17,43]. Figure 5b shows a dramatic suppression in the emission intensity at high Cr(VI) concentrations, and the fluorescence activity is completely quenched at 200 μM concentration. We further determined the limit of detection (LOD) of our sensor based on the quantitative analysis of the quenching efficiency using the Stern–Volmer equation: F_0_/F = 1 + K_sv_ [C] [44]. Here, F_0_ is the fluorescence intensity of the sensor without a droplet, F is the fluorescence intensity of the sensor with a droplet, C is the Cr(VI) concentration, and K_sv_ is the Stern–Volmer constant. The fluorescence intensity (F_0_/F) linearly increases with Cr(VI) concentration in the range of 1–60 μM, with a correlation coefficient of R^2^ = 0.95544 (see the inset in Figure 5b). We calculated the LOD using 3σ/K_sv_, where σ is the standard deviation. The LOD of our Cr(VI) sensor is 0.44 μM (i.e., 45.76 μg L^−1^), which indicates a high sensitivity.

We also investigated the selectivity of our Cr(VI) sensor using a series of metal cations (Ba^2+^, Ca^2+^, K^+^, Mg^2+^, Mn^2+^, Na^+^) and anions (F^−^, Br^−^, Cl^−^, I^−^, HSO_4_^−^ and CH_3_COO^−^) in aqueous droplets. Figure 5c,e show the UV-vis absorption spectra of aqueous droplets containing different metal cations and anions, respectively. The absorption band between 310 nm and 405 nm appears only with Cr(VI) and is not present with the metal cations and anions. Furthermore, the spectral overlap between the excitation of Eu^3+^ complex and absorption of Cr(VI) resulted in fluorescence quenching due to IFE enabling the detection of Cr(VI) in aqueous droplets. The fluorescent intensity decreased with increasing concentration of Cr(VI). Interestingly, the sensing mechanism could be proposed to be static quenching due to no effect on the fluorescence decay rates for the Cr(VI) fluorescent sensor (Table 1). Consequently, the presence of metal cations and anions in the aqueous droplet has no discernible effect on fluorescence quenching (Figure 5d,f) even at a high concentration (i.e., 0.1 M). This demonstrates the high selectivity of our Cr(VI) sensor even at high concentrations of metal cations and anions in aqueous droplets.

## 5. Conclusions

In conclusion, we developed a Eu^3+^ complex-based fluorescence sensor for Cr(VI) detection in water droplets. We fabricated our Cr(VI) sensor by electrospinning Eu^3+^ complex, PVDF, and silica particles. Furthermore, the spectral overlap between the excitation of Eu^3+^ complex and absorption of Cr(VI) resulted in fluorescence quenching due to IFE enabling the detection of Cr(VI) in aqueous droplets. Our Cr(VI) sensor displayed high sensitivity with a LOD of 0.44 μM (i.e., 45.76 μg L^−1^) and high selectivity. We anticipate that our results will open prospects for the design of novel inexpensive and fast Cr(VI) sensors.

## Figures and Tables

**Table 1:** Photophysical properties of the samples.

Sample	*τ_obs_* (μs)	*A_RAD_* (s^−1^)	*A_NR_* (s^−1^)	*Φ_Eu_* (%)	*Φ_tot_* (%)
FM 1	720.04	372.25	1011.5	26.90	66.02
FM 3	700.84	683.55	743.3	47.91	45.61
FM 4	726.32	628.44	760.4	45.25	33.74
FM 5	626.30	476.27	952.8	29.83	27.87

## Data Availability

The data that support the findings of this study are available from the corresponding author upon reasonable request.

## Supplementary Materials

The following supporting information can be downloaded at: https://www.mdpi.com/article/10.3390/nano13182574/s1, Section S1: Morphology of Silica Particles; Section S2: Cr(VI) Detection in Aqueous Droplets; Section S3: Surface Morphology and Water Repellency of Nanofibrous Membranes; Section S4: Repellency of Our Cr(VI) Sensor to Aqueous Droplets with Different Concentrations of Cr(VI); Section S5: Repellency of Our Cr(VI) Sensor to Aqueous Droplets with Different Metal Cations; Section S6: Repellency of Our Cr(VI) Sensor to Aqueous Droplets with Different Anions; Section S7: Energy Dispersive X-Ray Spectroscopy of Our Cr(VI) Sensor; Section S8: Fourier-Transform Infrared Spectroscopy (FTIR) of Nanofibrous Membranes; Section S9: Differential Scanning Calorimetry (DSC) of nanofibrous membranes; Section S10: X-Ray Diffraction (XRD).
