# Supplementary material for: Eu3+ Complex-Based Superhydrophobic Fluorescence Sensor for Cr(VI) Detection in Water
Source: Nanomaterials (Basel). 2023 Sep 17;13(18):2574. doi: 10.3390/nano13182574 (PMC10535327; doi:10.3390/nano13182574)
Supplement: Supplementary file 1 [file nanomaterials-13-02574-s001.zip › nanomaterials-2414426-supplementary.pdf]

nanomaterials

Supplementary Materials

Eu^{3+} Complex-Based Superhydrophobic Fluorescence Sensor for Cr(VI) Detection in Water

Wei Ding [1,†], Sravanthi Vallabhuneni [2,†], Jin Liu [1,†], Xinzhi Wang [1], Yue Zhao [1], Yao Wang [1], Qinglin Tang [1], Yanxin Wang [1], Xiaolin Zhang [1], Arun Kumar Kota [2,*] and Jianguo Tang [1,*]

Europium chloride ($EuCl_3 \cdot 6H_2O$) was purchased from Desheng Rare Earth Company, Ltd in Shandong (China). 2-thenoyltrifluoroacetone (greater and equal to 98.0% pure) and1,10-Phenanthroline (99% pure) were purchased from Aladdin (Shanghai, China).Ethanol (99.7%) was purchased from Tian in Fuyu Fine Chemicals Company, Ltd. (China). Other chemical reagents and metal chloride salts were purchased from Sinopharm Chemical Reagent Company, Ltd. (China). All the chemicals used were of analytical grade and used without further purification.

Section S1. Morphology of Silica Particles

The transmission electron micrographs (TEM) were obtained using a JEM-1200EX (JEOL Ltd., Japan). TEM imaging was conducted by drop casting the silica particle dispersion onto the copper grid and letting the solvent evaporate at room temperature. The TEM images (Figure S1) show that the synthesized silica particles were spherical with a size of ~400 nm.

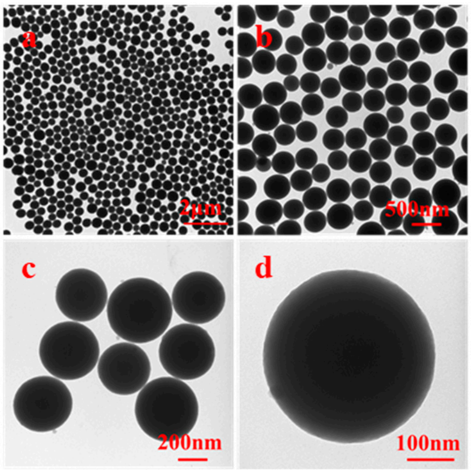

Figure S1. TEM images of silica particles synthesized via Stöber method.

Section S2. Cr(VI) Detection in Aqueous Droplets

Cr(VI) detection in aqueous droplets was conducted using fluorescence spectra obtained from micro-spectroscopy. Figure S2 shows a schematic depicting the collection of fluorescence spectra on our Cr(VI) sensor using micro-spectroscopy.

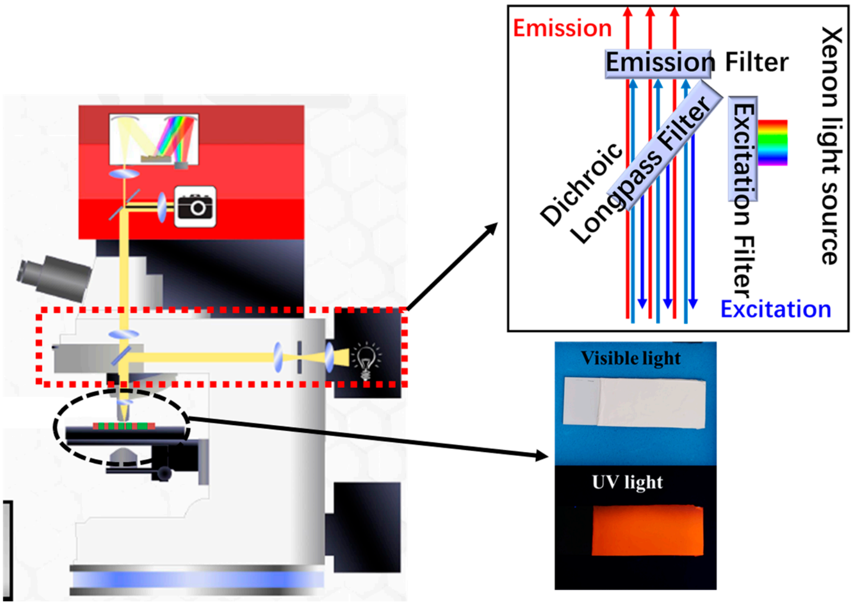

Figure S2. Schematic depicting the collection of fluorescence spectra on Cr(VI) sensor.

Section S3. Surface Morphology and Water Repellency of Nanofibrous Membranes

Figure S3 shows the nanofibrous morphology and static contact angles (CA) of a 3 mL water droplet on FM 2 (PVDF only), FM 3 (PVDF and Eu^{3+} complex) and FM 4 (PVDF, Eu^{3+} complex and silica particles) nanofibrous membranes.

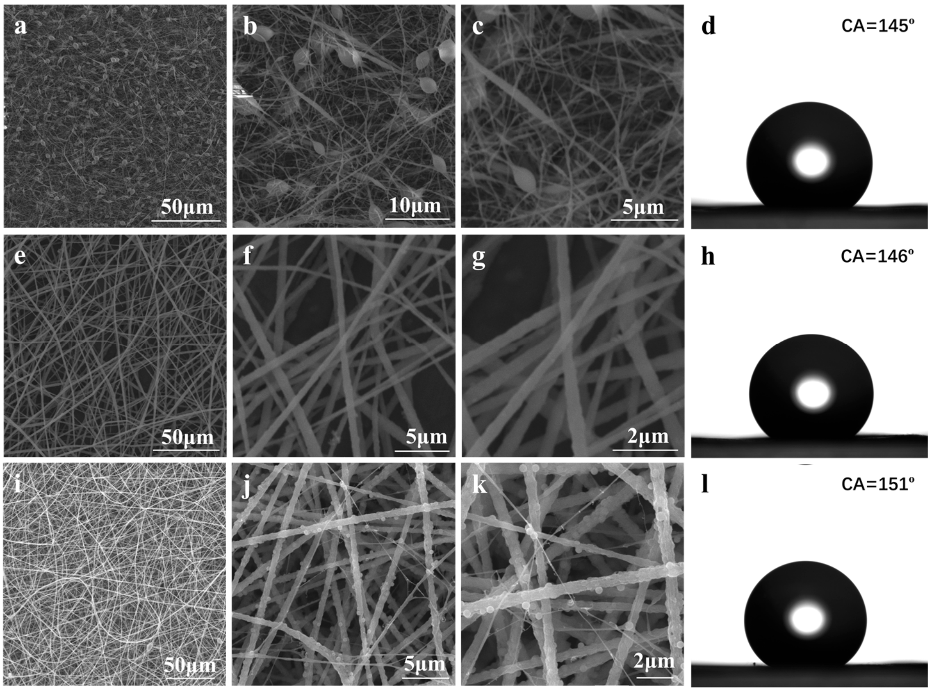

Figure S3. SEM images and contact angles of water droplet on FM 2, FM 3 and FM 4. (**a**–**c**) SEM images of FM 2 at different magnifications. (**d**) Contact angle of water droplet on FM 2. (**e**–**g**) SEM images of FM 3 at different magnifications. (**h**) Contact angle of water droplet on FM 3. (**i**–**k**) SEM images of FM 4 at different magnifications. (**l**) Contact angle of water droplet on FM 4.

Section S4. Repellency of Our Cr(VI) Sensor to Aqueous Droplets with Different Concentrations of Cr(VI)

Figure S4 shows that our Cr(VI) sensor (i.e., FM 5) is extremely repellent to aqueous droplets containing Cr(VI) at different concentrations.

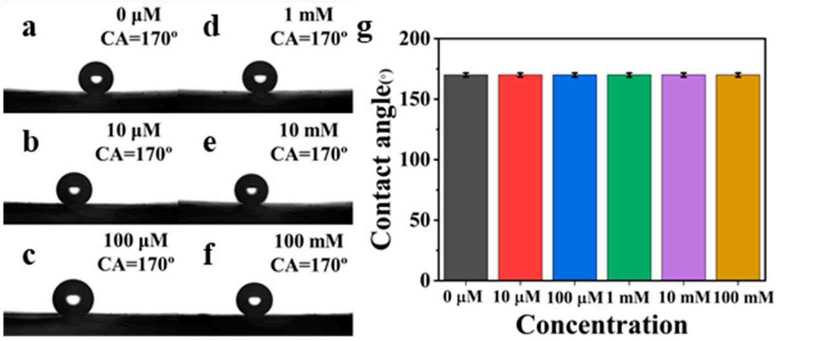

Figure S4. Repellency of our Cr(VI) sensor to aqueous droplets with different Cr(VI) concentrations. (**a**–**f**) Static contact angles of 3 mL water droplets containing Cr(VI) at 0 mM, 10 mM, 100 mM, 1 mM, 10 mM, and 100 mM concentrations, respectively on our Cr(VI) sensor. (**g**) Contact angle as a function of Cr(VI) concentration in aqueous droplets. Error bars indicate standard deviation.

Section S5. Repellency of our Cr(VI) Sensor to Aqueous Droplets with Different Metal Cations.

Figure S5 shows that our Cr(VI) sensor is extremely repellent to aqueous droplets containing different metal cations.

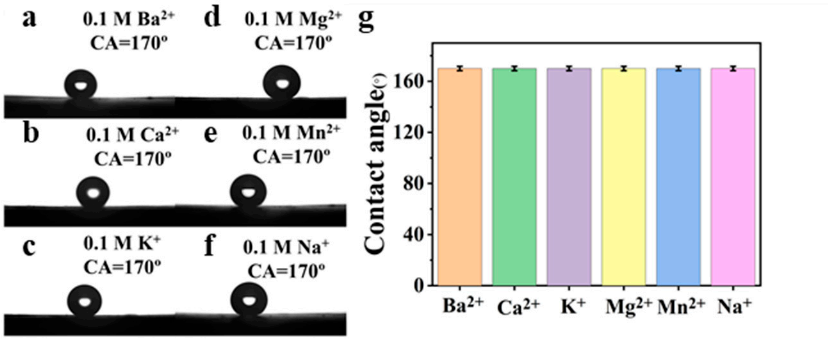

Figure S5. Repellency of our Cr(VI) sensor to aqueous droplets with different metal cations. (**a**–**f**) Static contact angles of 3 mL water droplets containing Ba^{2+}, Ca^{2+}, K^{+}, Mg^{2+}, Mn^{2+}, and Na^{2+} at 0.1 M concentration, respectively on Cr(VI) sensor. (**g**) Contact angle of aqueous droplets with different metal cations. Error bars indicate standard deviation.

Section S6. Repellency of Our Cr(VI) Sensor to Aqueous Droplets with Different Anions

Figure S6 shows that our Cr(VI) sensor is extremely repellent to aqueous droplets containing different anions.

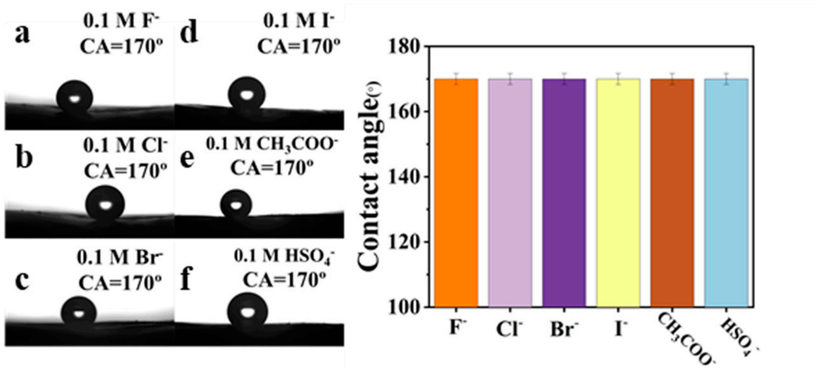

Figure S6. Repellency of our Cr(VI) sensor to aqueous droplets with different anions. (**a–f**) Static contact angles of 3 mL water droplets containing F^-, Cl^-, Br^-, I^-, CH_3COO^-, and HSO_4^- at 0.1 M concentration, respectively on Cr(VI) sensor. (**g**) Contact angle of aqueous droplets with different anions. Error bars indicate standard deviation.

Section S7. Energy Dispersive X-Ray Spectroscopy of Our CR(VI) Sensor

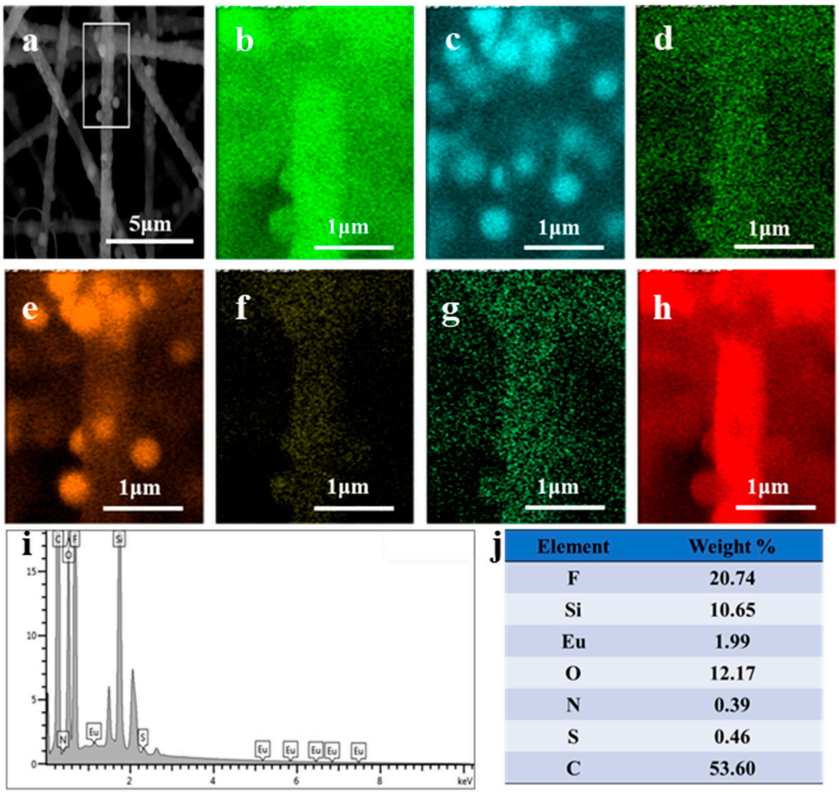

Figure S7. EDS elemental mapping of FM 5. (**a**) SEM image showing the location of EDS mapping on Cr(VI) senor. (**b–h**) EDS elemental maps of F, Si, Eu, O, N, S, and C, respectively. (**i,j**) The weight percentage of the elements in the sensor.

Section S8. Fourier-Transform Infrared Spectroscopy (FTIR) of Nanofibrous Membranes

The composition of nanofibrous membranes was characterized using Fourier-transform infrared (FTIR) spectroscopy (Thermo Electron Scientific Instruments Corp., Nicolet 5700, USA) in the range of 4000-400 cm^{-1} in ATR mode. Fig. S8 shows the FTIR spectra of FM 2, FM 3, FM 4, and FM 5. The bending and wagging vibrations of $-CF_2$ group at 491 cm^{-1} and 614 cm^{-1} correspond to a-PVDF polymorph. The vibration bands at 445 cm^{-1} correspond to b-phase parallel to a-axis. The bands at 840 cm^{-1} and 510 cm^{-1} represent $-CH_2$ rocking mode and $-CF_2$ bending mode, respectively. The characteristic bands at 840 cm^{-1},

510 cm^{-1}, and 1275 cm^{-1} correspond to b-phase. The bands at 1401 cm^{-1} and 1075 cm^{-1} represent $-CH_2$ wagging. The band at 1100 cm^{-1} represent Si-O group. The characteristic bands at 720 cm^{-1}, 1158 cm^{-1}, 1450 cm^{-1}, and 1240 cm^{-1} correspond to $-CH_2$, -CF, $-CF_2$, and $-CF_3$, respectively.

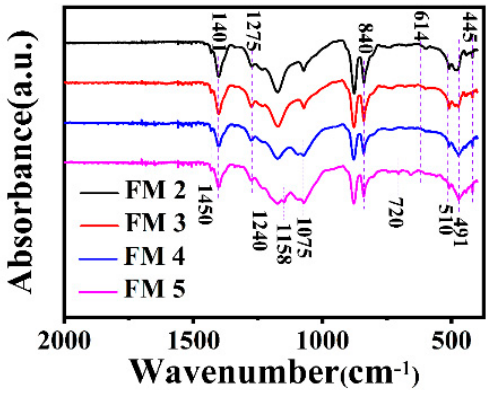

Figure S8. FTIR spectra of FM 2, FM 3, FM 4 and FM 5.

Section S9. Differential Scanning Calorimetry (DSC) of Nanofibrous Membranes

DSC analysis was used to determine the melting temperature and the heat of fusion of the nanofibrous membranes (Q20, Waters LCC). The samples were scanned from 40-200°C at a rate of 10 °C min^{-1} under 50mL min^{-1} N_2 gas flow. The DSC curves (Figure S9) of nanofibrous membranes (FM 2, FM 3, FM 4, FM 5) indicate that there is no change in the phase and melt temperatures (represented by endothermal peak at 167°C) due to the incorporation of silica particles and Eu^{3+} complex.

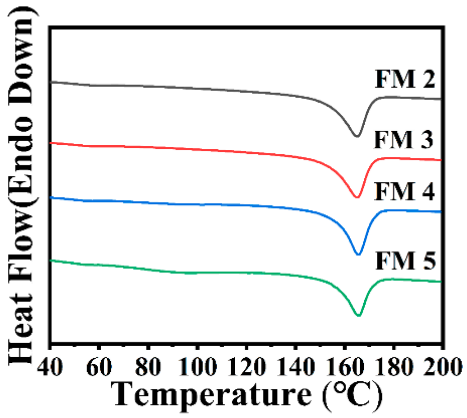

Figure S9. DSC curves of FM 2, FM 3, FM 4 and FM 5.

Section S10. X-Ray Diffraction (XRD)

The phases of PVDF were characterized using XRD (XD3, Beijing Purkinje General Instrument Co., Ltd.) in the range of 0 to 80° with a step size of 0.02, using a Cu Kα radiation (λ = 0.154 nm) source. Fig. S10 shows the XRD patterns of the Cr(VI) sensor. The peaks at 14.7° and 24.8° correspond to (210) and (321) planes of silica particles, respectively. The peaks at 20.7° and 20.8° correspond to (110) plane of a-PVDF, and (200) or (110) plane of b-PVDF, respectively.

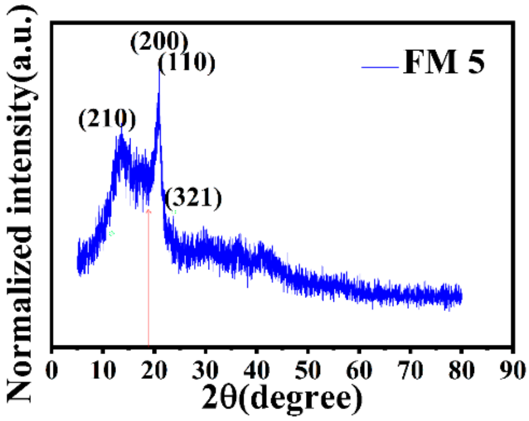

Figure S10. XRD patterns of our Cr(VI) sensor.
